# A Practical Treatise on Sexual Disorders of the Male and Female

**Published:** 1898-02

**Authors:** 


### Book Reviews.

---

A PRACTICAL TREATISE ON SEXUAL DISORDERS OF THE MALE AND FEMALE. By ROBERT W. TAYLOR, M. D., Clinical Professor of Venereal Diseases in the College of Physicians and Surgeons, New York. Octavo, 148 pages, with seventy-three illustrations and eight plates in color and monochrome. New York and Philadelphia: Lea Brothers & Co. 1897.

The author alleges in his preface that his aim is to portray sexual disorders on the basis of advanced knowledge of the anatomy, physiology and pathology of the sexual system. Heretofore, functional disturbances, sensory and motor neuroses—symptoms of sexual debility—have been inefficiently, erroneously and incompletely presented. Within the past few years the study of sexual and urinary diseases has been directed to a more advanced knowledge of the functions, structures and pathology of the genito-urinary system. The author states that his presentation of sexual disorders has been largely elaborated from information founded upon personal investigation and experience, and that the basis of the study of genito-urinary diseases will be so established as to be found helpful to the general practitioner in the management of a large group of cases which have been ill-understood and even avoided by the every-day medical man.

Chapter I.—In this chapter the anatomy of the sexual organs is carefully and accurately presented, including some new and original illustrations. In describing the nerves of the penis, it is stated that the sensorium commune of the external male genitals is seated in the glands, and that there is also an internal sensorium located in the middle of the prostatic urethra. The ducts of three orders of muciperous glands open into the anterior urethra. These are the glands of Littré, Morgagni and Cowper. The secretions of Littré's follicles and Morgagni's crypts, lubricate and alkalinise the urethral canal, and thus prepare the way for spermatozoa, while the secretion from Cowper's glands forms part of the seminal discharge.

The prostate is essentially a sexual gland, incidentally employed in urinating, but its chief function is sexual. To support its glandular formation there is fibrous connective tissue and unstriped muscular substance. Surrounding the gland are bundles of unstriped muscular fibers, which expel its secretion into the urethra. In the young the prostate is made up of groups of fairly well defined glandular lobules. The gland ducts are not invested with muscular structure and there is no storage for its secretion, which is copiously elaborated and expelled during the sexual act. [The prostatic secretion probably supplies the greater bulk of the seminal fluid.]

The seminal vesicles are blind-ending tubes with diverticula of various sizes. The vesicle is two and a half inches long, one-half to one inch wide and one-quarter to one-half thick. Columnar and cuboidal epithelium line these vesicles and the mucous membrane is studded with the orifices of mucous tubular glands and lays in folds, thus greatly increasing its extent. The vesicular walls are thick and trabeculated and contain abundant muscular tissue.

The author takes issue with the general belief that the seminal vesicles are reservoirs for storing the spermatozoa and that their secretion fructifies and enlivens the spermatic cells. He refers to the researches of Dr. Huntington, who is convinced that the semen [spermatozoa ?] never reaches the seminal vesicles and that their presence in these organs is the result of accidental conditions.

The author alleges that there is no structural mechanism and no known physiological process that could pass the spermatozoa into the vesicles.

Chapter II. relates to the physiology of the male sexual function. Chapter III., Fig. 16, shows the normal secretion of the seminal vesicles loaded with spermatozoa. [How did they get there ? Did they climb in out of respect for the opinion of Dr. Fuller ?] The author says it is doubtful whether there is a special nerve center for ejaculation. Fuller says that a so-called sexual nerve center in the lumbar spine has the chief control of ejaculation.

The erotic impression and erection of the penis arouses the testicles into increased functional activity. During sexual erethism the testicles are drawn up to the internal abdominal rings, and synchronously semen [spermatozoa ?] escapes from the coni vasculosi and reaches the vasa, carried forward by the powerful rhythmical contraction of the strong muscular fibers surrounding these tubes, and that they are forced into the ampulla, which, becoming distended, causes rhythmic contraction synchronously with the seminal vesicle, and the contents of these structures meet for the first time in the ejaculatory ducts.

It is estimated that there are from 250,000,000 to 400,000,000 spermatozoa thrown out with every ejaculation. Such a rush must cause crowding in the bristle-sized lumen of the vasa deferentia.

The elaboration by the testicles of such highly organised product as spermatozoa and their passage through the coni vasculosi, vasa, ejaculatory ducts and urethra would seem to require more time than the ordinary sexual act. The views of Fuller and Taylor are widely at variance, and both should be read to be appreciated. The question is, do the seminal vesicles store the spermatozoa ?

The semen is a composite liquid, made up of the combined secretions of the testicles, seminal vesicles, prostate gland, Cowper's glands and the muciporous glands of the urethra.

Impotence in the male is divided into four classes : (1) Psychical ; (2) symptomatic ; (3) atonic ; (4) organic.

*Epididymitis.*—As a result of bilateral gonorrheal epididymitis, a man may be sterile without losing his virility.

*Conclusions.*—(1) In all cases of unilateral epididymitis treatment should not cease with the decline of the acute stage ; (2) bilateral epididymitis should receive energetic and long-continued treatment ; (3) prognosis in recent cases is very favorable for a perfect cure ; orchitis as a complication of gonorrheal epididymitis is rare and usually quickly disappears.

Chapter XVI.—Chronic inflammation of the bulbous and prostatic urethra. The gonorrheal process in the bulbous urethra is very chronic, persistent and rebellious. There may be no visible secretion or it may be very copious. Its earlier stage is characterised by a slight discharge, threads and uneasy or burning sensation. The bulbous urethra, owing to its different anatomical formation and environment, is often profoundly affected by the gonorrheal process. Inodular stricture forms sooner or later and the parts become rigid and stenotic ; urination is obstructed, and the sexual function impaired or lost.

Chronic posterior urethritis may exist for five or twenty years, unknown, as a gonorrheal sequelæ, to its possessor and unrecognised by his physician. Its victim is subject to exacerbations of urethritis, following excesses, and as these recrudescences readily subside, little or no attention is given to it until sexual weakness, hematuria or some other prominent symptom attracts attention.

For local treatment deep injections are applied after the manner proposed by Ultzman, and if the case is very rebellious thirty to sixty grains of nitrate of silver to the ounce of water is applied through the endoscope with a porte remède.

The following chapters relate to various other maladies which afflict the genital system.

This volume is illustrated by some new and original cuts, the personal work of the author, whose resources are fortified by great experience and close observation.

It is a fine specimen of the printer's art. B. H. D.

